# Adaptation to Industrial Stressors Through Genomic and Transcriptional Plasticity in a Bioethanol Producing Fission Yeast Isolate

**DOI:** 10.1534/g3.119.400986

**Published:** 2020-02-21

**Authors:** Dane Vassiliadis, Koon Ho Wong, Jo Blinco, Geoff Dumsday, Alex Andrianopoulos, Brendon Monahan

**Affiliations:** * Genetics, Genomics & Systems Biology, School of Biosciences, The University of Melbourne, Parkville, Victoria, Australia; † Commonwealth Scientific and Industrial Research Organisation (CSIRO), Parkville, Victoria, Australia; ‡ Faculty of Health Sciences, University of Macau, Macau SAR, China; § Institute of Translational Medicine, University of Macau, Macau SAR, China; ** Wilmar Ltd. Mackay, Queensland, Australia; †† Cancer Therapeutics (CTx), Parkville, Victoria, Australia

**Keywords:** Schizosaccharomyces pombe, bioethanol, transcriptional adaptation, genomic plasticity

## Abstract

*Schizosaccharomyces pombe* is a model unicellular eukaryote with ties to the basic research, oenology and industrial biotechnology sectors. While most investigations into *S. pombe* cell biology utilize Leupold’s 972*h*^-^ laboratory strain background, recent studies have described a wealth of genetic and phenotypic diversity within wild populations of *S. pombe* including stress resistance phenotypes which may be of interest to industry. Here we describe the genomic and transcriptomic characterization of Wilmar-P, an *S. pombe* isolate used for bioethanol production from sugarcane molasses at industrial scale. Novel sequences present in Wilmar-P but not in the laboratory *S. pombe* genome included multiple coding sequences with near-perfect nucleotide identity to *Schizosaccharomyces octosporus* sequences. Wilmar-P also contained a ∼100kb duplication in the right arm of chromosome III, a region harboring *ght5*^+^, the predominant hexose transporter encoding gene. Transcriptomic analysis of Wilmar-P grown in molasses revealed strong downregulation of core environmental stress response genes and upregulation of hexose transporters and drug efflux pumps compared to laboratory *S. pombe*. Finally, examination of the regulatory network of Scr1, which is involved in the regulation of several genes differentially expressed on molasses, revealed expanded binding of this transcription factor in Wilmar-P compared to laboratory *S. pombe* in the molasses condition. Together our results point to both genomic plasticity and transcriptomic adaptation as mechanisms driving phenotypic adaptation of Wilmar-P to the molasses environment and therefore adds to our understanding of genetic diversity within industrial fission yeast strains and the capacity of this strain for commercial scale bioethanol production.

Increasing global attention is being focused toward the production of alternative sources of renewable energy to combat declining global fossil fuel reserves and the threat of environmental catastrophe brought about by climate change. Bioethanol is a renewable alternative to petroleum which is compatible with most current vehicles and has reduced environmental impact. Bioethanol is produced primarily through the fermentation of sugars by yeast species, particularly *Saccharomyces cerevisiae* (Mohd Azhar *et al.* 2017). Whole-genome sequencing approaches are increasingly being applied to yeast genomes, providing the opportunity for comparative genomic studies on a global scale (Piškur and Langkjær 2004). Several such studies have revealed extensive genetic diversity present within the genomes of *S. cerevisiae* wine, beer, sake, bread and bioethanol producing strains, as well as naturally occurring “wild” isolates from around the world (Yue *et al.* 2017; Gallone *et al.* 2016; Peter *et al.* 2018; Liti *et al.* 2009). Additional studies have revealed extensive genetic variation within currently utilized *S. cerevisiae* industrial bioethanol strains, including cross-hybridization, horizontal gene transfer between *S. cerevisiae* and other *Saccharomyces* species, and multiple genomic rearrangement events (Argueso *et al.* 2009; Babrzadeh *et al.* 2012; Li *et al.* 2014; McIlwain *et al.* 2016).

Stress factors and inhibitory compounds limit the efficiency of industrial scale fermentation by yeasts (Deparis *et al.* 2017). Common stressors during industrial fermentations include temperature, pH, osmotic and ethanol stress (Zheng and Wang 2015). The presence of contaminating organisms or toxins often found in more complex feedstocks such as molasses or lignocellulosic substrates can also impose stress on the industrial organism and inhibit its growth and/or fermentation capacity (Dorta *et al.* 2005). Continued passaging through an environment under artificial selection has been shown to select for industrial strains that can better withstand such pressures, or that possess improved fermentation characteristics (Çakar *et al.* 2012; Mans *et al.* 2018). Certain wild or industrially domesticated *S. cerevisiae* strains have been shown to possess stress tolerance phenotypes or improved fermentation performance when compared to the laboratory type strain S228c (Borneman *et al.* 2013b; Borneman *et al.* 2013a; Steensels and Verstrepen 2014; Steensels *et al.* 2014). Therefore, understanding the genetic mechanisms underlying these phenotypic differences could guide the construction of strains which exhibit improved industrial performance.

The fission yeast *Schizosaccharomyces pombe* is widely studied as a model of eukaryotic cell biology. While much of this work has been conducted in the genetic background of Leupold’s 972*h*^-^ laboratory strain (hereafter “laboratory *S. pombe*”) (Hoffman *et al.* 2015; Fantes and Hoffman 2016), non-laboratory strains of *S. pombe* are also utilized in industrial fermentation processes, including bioethanol production and winemaking (Benito 2019; Benito *et al.* 2016; Choi *et al.* 2010). Recent studies have highlighted extensive genetic and structural variation within geographically isolated *S. pombe* populations (Jeffares *et al.* 2015; Jeffares *et al.* 2017; Brown *et al.* 2011) however, few studies have examined the genetic features of *S. pombe* strains that have been harnessed for industrial purposes. In Queensland, Australia, a wild isolate of *S. pombe*, Wilmar-A, was adapted for the industrial-scale fermentation of sugarcane molasses into bioethanol. Repeated passaging of Wilmar-A through industrial grade molasses resulted in the isolation of the Wilmar-P strain, which is currently used for industrial scale bioethanol production.

In this study we combined whole genome, whole transcriptome and chromatin immunoprecipitation (ChIP) sequencing approaches to develop a comprehensive understanding of the Wilmar-P genomic and transcriptomic landscape compared to laboratory *S. pombe*. We identified extensive sequence level polymorphism comparable to previous studies of wild *S. pombe* isolates and multiple instances of structural variation, particularly at chromosome II subtelomeres. DNA unique to the Wilmar strain genome contained protein-coding sequences with near perfect nucleotide identity to those found in the related *Schizosaccharomyces octosporus* genome. These genes are expressed and form part of the genetic reportoire of Wilmar-P. Transcriptomic analysis revealed the adaptation of a transcriptional program which is active during growth in molasses and results in suppression of core environmental stress response and sexual differentiation pathway gene expression while promoting expression of hexose transporters and transmembrane efflux pumps. Finally we examined the regulatory network of Scr1, a transcriptional repressor of carbon source responsive genes, and found that it is involved in the regulation of multiple genes differentially expressed in Wilmar-P on molasses. An expanded number of Wilmar-P Scr1 targets were identified compared to laboratory *S. pombe* Scr1 on molasses, and Wilmar-P Scr1 binding was associated with transcriptional upregulation at multiple loci suggesting that Scr1 function is repurposed in Wilmar-P. Together, these data highlight both genomic plasticity and transcriptional adaptation as mechanisms by which *S. pombe* adapts to industrial environments.

## Materials and Methods

### Strains, media and growth assays

Strains used in this study are listed in Table S1. Strains were cultured on yeast extract medium (YES) with supplements and carbon sources as described in the text (Sabatinos and Forsburg 2010). Molasses medium contained 22.2% (v/v) Wilmar molasses, 0.6% (w/v) ammonium sulfate, 0.4% (w/v) potassium dihydrogen orthophosphate and was buffered to pH 4.2-4.5 with sulphuric acid (Wilmar Bioethanol, *pers. comm*.).

### Pulsed field gel electrophoresis (PFGE)

Chromosomal plugs were generated from laboratory *S. pombe*, Wilmar-A and Wilmar-P as described previously (Brown *et al.* 1990; Brown 1988). Chromosomes were separated on a CHEF DRIII system (BioRad) using 0.8% (w/v) certified megabase agarose gels. Commercial laboratory *S. pombe* plugs (Biorad) were used as a chromosome size standard. Electrophoresis was conducted over 48 hr with an applied current of 2V/cm (90V total) and a 20 to 30 min ramp gradient switch time at an angle of 106°. 1xTAE buffer (Sambrook and Russell 2001) was circulated at 14°.

### Purification and sequencing of nucleic acids

*S. pombe* genomic DNA was purified using the phenol-chloroform method as described previously (Sabatinos and Forsburg 2010). High molecular weight genomic DNA for Pacific Biosciences (PacBio) sequencing was purified by spheroplasting cells with Zymolyase 20T (Nacalai Tesque) before phenol-chloroform extraction without vortexing. Whole genome sequencing (WGS) Illumina libraries were constructed by Macrogen (South Korea) and sequenced using an Illumina HiSeq 2000. PacBio library construction was performed at the Doherty Applied Microbial Genomics facility (Melbourne, Australia) using an average library size of 12kb and sequenced using a Pacific Biosciences RSII. RNA-seq libraries were generated by the Australian Genomic Research Facility (AGRF, Melbourne, Australia) using Illumina TruSeq protocols and sequenced on an Illumina HiSeq 2500.

### Wilmar strain de novo genome assembly, annotation and alignment

PacBio reads derived from the Wilmar-P strain were assembled using Canu v1.7 (Koren *et al.* 2017). Illumina reads were then aligned to the draft assembly using BWA v0.7.17 (Li 2013) and Pilon v1.23 was used to correct sequencing errors and polish the assembly (Walker *et al.* 2014). Genome annotations were transferred from the *S. pombe* reference library using the Rapid Annotation Transfer Tool (RATT) v1.0 (Otto *et al.* 2011). Additional genome annotation was performed using Maker v2.31.1 (Cantarel *et al.* 2008). Identical annotations from RATT and Maker were merged. For gene loss studies, Wilmar isolate Illumina reads were mapped to the laboratory *S. pombe* reference genome (ASM294 v2.20) using Bowtie2 v2.3.4.1 (Langmead and Salzberg 2012) with the following settings:–sensitive –minins 0 –maxins 700 and read coverage per base was determined using bedtools v2.27.1. Genome *de novo* assemblies were aligned using nucmer v3.1(Kurtz *et al.* 2004). Open reading frames (ORFs) were identified using OrfM v0.7.1 (Woodcroft *et al.* 2016) and characterized using NCBI BLASTn and BLASTx.

### Genome copy number analysis

Copy number variants were called using cnvkit (Talevich *et al.* 2016) with a bin size of 100bp and assuming a ploidy of 1. For global examination of copy number reference genome coverage was calculated at each position using bedtools v2.27.1 (Quinlan and Hall 2010), and coverage graphs were generated by averaging the read coverage within sliding windows (10kb window, 1kb step) across each chromosome using a custom Python script. Genes overlapping regions of copy number gain or loss by cnvkit were verified by examining the raw reads mapped to the laboratory *S. pombe* genome using the integrative genomics viewer.

### Sequence level analysis

Single nucleotide polymorphisms, multi-nucleotide variants and insertions-deletions (SNPs, MNVs and INDELs) were called from the mapped Illumina reads using freebayes and the Genome Analysis Toolkit (GATK) Unified Genotyper v3.6-0 (DePristo *et al.* 2011; Garrison and Marth 2012) with the sample ploidy set to 1 (haploid). Variants were annotated using snpEff v4.3b (Cingolani *et al.* 2012b) and the variant effect predictor (VEP) (McLaren *et al.* 2016). Quality > 30 and depth of sequencing > 10 filters were applied using snpSift v4.3b (Cingolani *et al.* 2012a). Raw whole genome sequence data from previously sequenced *S. pombe* isolates (Jeffares *et al.* 2015) were downloaded from the European Nucleotide Archive (ENA, accession code: PRJEB2733 and PRJEB6284). Variants from all strains were merged using VCF-tools v0.1.15 (Danecek *et al.* 2011). SNPrelate v1.16.0 (Zheng *et al.* 2012) was used to analyze relatedness of the Wilmar isolates *vs.* the strains in the Jeffares *et al.* study (Jeffares *et al.* 2015) and generate a phylogeny based on hierarchical clustering of SNP variation among the strains. Except for a linkage disequilibrium cutoff score of 0.1, default settings for SNPRelate were used.

### Wilmar-P Scr1 TAP tagging, chromatin immunoprecipitation (ChIP) and ChIP-seq library preparation

A tandem affinity purification (TAP) tag was fused to the 3′ end of the Wilmar-P *scr1^+^* coding sequence using methods described previously (Bähler *et al.* 1998). ChIP was performed as described previously (Monahan *et al.* 2008) using two independent biological replicates for each strain and experimental condition. To prepare chromatin for ChIP, cells cultured in YES 3% (w/v) glucose to OD_595_ ≈ 0.5 were harvested by centrifugation, washed twice in sterile water, and split evenly across 50mL YES 3% (w/v) glucose, or molasses medium. After a further 4 hr of culturing, cells were crosslinked with 1% (v/v) formaldehyde for 15min at room temperature with mild shaking. Crosslinking was quenched with 2.5mL 2.5M glycine and cells were lysed by bead beating. The chromatin fraction was sonicated to 200-500bp fragments using a Bioruptor sonicator (Diagenode) on high power using 5x 9 min cycles of 30 sec ON, 60 sec OFF. For TAP-tag ChIP, 4uL mouse anti-protein A antibody (Sigma P2921) was coupled to 100uL pan-mouse Dynabeads (Invitrogen). For RNA PolII^Ser5^ ChIP, 5uL of mouse anti-RNA Polymerase II^Ser5^ phospho-CTD antibody (clone 3E8, Merck 04-1572) was coupled to 100uL goat anti-mouse Dynabeads (Invitrogen) to saturate the beads with antibody and prevent cross-reactivity with the Protein A moiety of the TAP tag. Immunoprecipitated DNA was quantified using a high-sensitivity DNA assay on a Qubit 2.0 fluorometer (Invitrogen). ChIP-seq library preparation was performed as described previously (Wong *et al.* 2013). Library DNA was examined for quality on an Agilent Bioanalyser 2100 using DNA high-sensitivity chips. ChIP-seq DNA was sequenced on an Illumina HiSeq 2500 using either a 75bp single-end or 100bp paired-end strategy.

### RNA-seq and ChIP-seq analysis

Laboratory *S. pombe* RNA-seq and ChIP-seq data for laboratory media growth conditions from our previous study (Vassiliadis *et al.* 2019) was downloaded from the Sequence Read Archive (GSE123617 and GSE123614). Illumina adapter sequences were removed with Trim_Galore! v0.4.3, a wrapper for Cutadapt, using default parameters (Krueger 2016; Martin 2011). To examine Wilmar specific gene expression, Wilmar-A and Wilmar-P strain RNA-seq reads were aligned to their respective *de novo* assembled genomes using Tophat2 v2.1.1 (Kim *et al.* 2013) using the following parameters:–max-intron-size 5000 –library-type fr-firststrand. Wilmar-A, Wilmar-P and laboratory *S. pombe* reads were also mapped to the *S. pombe* reference genome (ASM294v2.25) using the same conditions. FeatureCounts v1.5.2 (Liao *et al.* 2014) was used to count reads mapping to genomic features using the following parameters: -O -T 4. For stranded libraries, the -s 2 parameter was set. Differential gene expression analysis was performed using DESeq2 v1.16.1 with library strandedness incorporated as a factor in the generalized linear model (Love *et al.* 2014). Genes with an absolute log2 fold change (Log_2_FC) greater than 1 and a False Discovery Rate (FDR) adjusted p-value less than 0.05 were considered differentially expressed. ChIP-seq peak detection was performed using a workflow based on MACS v2.1.1 (Zhang *et al.* 2008) coupled with irreproducible discovery rate (IDR) correction across replicates as described previously (Landt *et al.* 2012). An input sample was used to provide background enrichment correction for each condition. Deeptools2 v2.5.3 (Ramírez *et al.* 2016) and ChIPseeker (Yu *et al.* 2015) were used for visualization of input normalized ChIP-seq samples. Gene ontology and gene set enrichment analyses were performed using AnGeLi with default parameters (Bitton *et al.* 2015) or clusterProfiler (Yu *et al.* 2012).

### Data availability

Whole genome sequencing datasets generated in this study have been deposited in the NCBI Sequence Read Archive (SRA) under study accession PRJNA594219. RNA-seq and ChIP-seq datasets have been deposited in the NCBI Gene Expression Omnibus (GEO) under study accessions GSE141715 (RNA-seq) and GSE141744 (ChIP-seq). File S1 contains supplementary figures. File S2 contains supplementary tables. Supplemental material available at figshare: https://doi.org/10.25387/g3.11798448.

## Results

### The Wilmar isolate is resistant to nutrient and temperature stress

**Figure 1:**
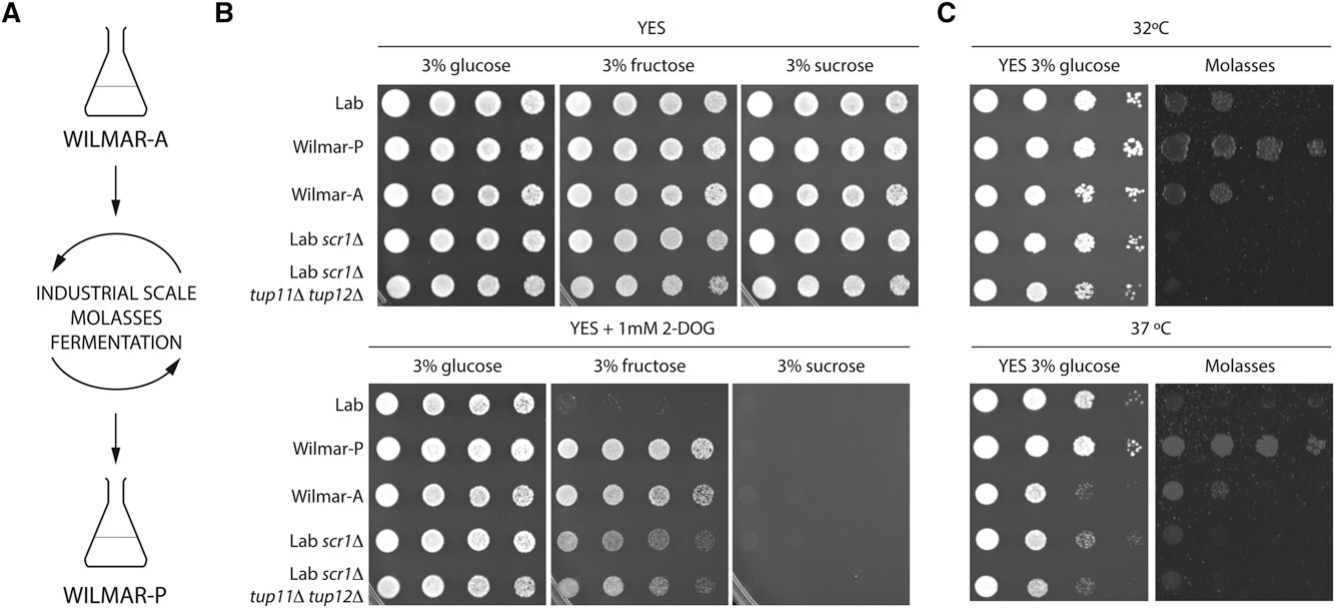
Wilmar-P exhibits superior growth in 2-DOG, high temperature and molasses conditions. A) Schematic showing the genesis of Wilmar-P following repeated passaging of Wilmar-A through the industrial molasses fermentation environment. B) Spot plate dilution assays on the indicated media were incubated at 32°C for 48-72h unless indicated otherwise. C) Spot plate dilution assays on the indicated media were incubated at the indicated temperature for 48-72h. Images are representative of three biological replicates. Lab = *S. pombe* 972*h*^-^. YES = Yeast extract + supplements medium. 2-DOG = 2-deoxy-glucose.

The Wilmar production isolate (Wilmar-P) was derived from an ancestral wild *S. pombe* strain (Wilmar-A) that was adapted for industrial use via repeated passaging through molasses fermentations (Figure 1A). Given that carbon utilization is key to bioethanol production (Mohd Azhar *et al.* 2017), we examined differences in the growth patterns of Wilmar-A and Wilmar-P to laboratory *S. pombe* and to mutants deficient in carbon catabolite repression (CCR), the predominant carbon-responsive transcriptional regulatory mechanism in *S. pombe* (Vassiliadis *et al.* 2019). Wilmar-P and *scr1*Δ showed slightly improved growth compared to laboratory *S. pombe*, Wilmar-A and *scr1*Δ *tup11*Δ *tup12*Δ strains when cultured at the optimal growth temperature of 32° (Figure 1B). When cultured in the presence of the metabolically inert glucose analog 2-deoxyglucose (2-DOG), growth of all strains was normal on glucose + 2-DOG but completely inhibited on sucrose + 2-DOG (Figure 1B). On fructose + 2-DOG media, growth of Wilmar-A and Wilmar-P was only slightly affected whereas growth of laboratory *S. pombe* was completely inhibited. The *scr1*Δ and *scr1*Δ *tup11*Δ *tup12*Δ strains (hereafter “CCR mutants”) also showed poorer growth compared to the Wilmar isolates on 2-DOG containing medium. We next cultured these strains on molasses medium, mimicking the industrial environment. At 32° vastly improved growth of Wilmar-P was observed compared to Wilmar-A and laboratory *S. pombe* consistent with the adaptation of this strain to the molasses fermentation environment (Figure 1C). In contrast, the CCR mutants showed no appreciable growth on molasses. Given that sucrose is the primary carbon source within molasses (Jain and Venkatasubramanian 2017; Wythes *et al.* 1978) and the CCR mutants are derepressed for *inv1*^+^, which encodes the predominant sucrose invertase in *S. pombe* (Tanaka *et al.* 1998; Vassiliadis *et al.* 2019), this result suggests that factors other than sucrose metabolism are more important for successful growth on molasses. To simulate heat stress conditions which can occur during industrial fermentations, we repeated the experiment at 37°. The molasses growth phenotypes of Wilmar-P and Wilmar-A were maintained at the higher temperature while the growth of laboratory *S. pombe* and the CCR mutants were further impaired on both YES + glucose and molasses medium (Figure 1C). Together, these results show that the Wilmar background is resistant to 2-DOG and additionally, that Wilmar-P possesses superior growth on molasses and improved temperature stress tolerance compared to Wilmar-A and laboratory *S. pombe*.

### Non-synonymous mutations affect almost half of the genes in the Wilmar strain

**Figure 2:**
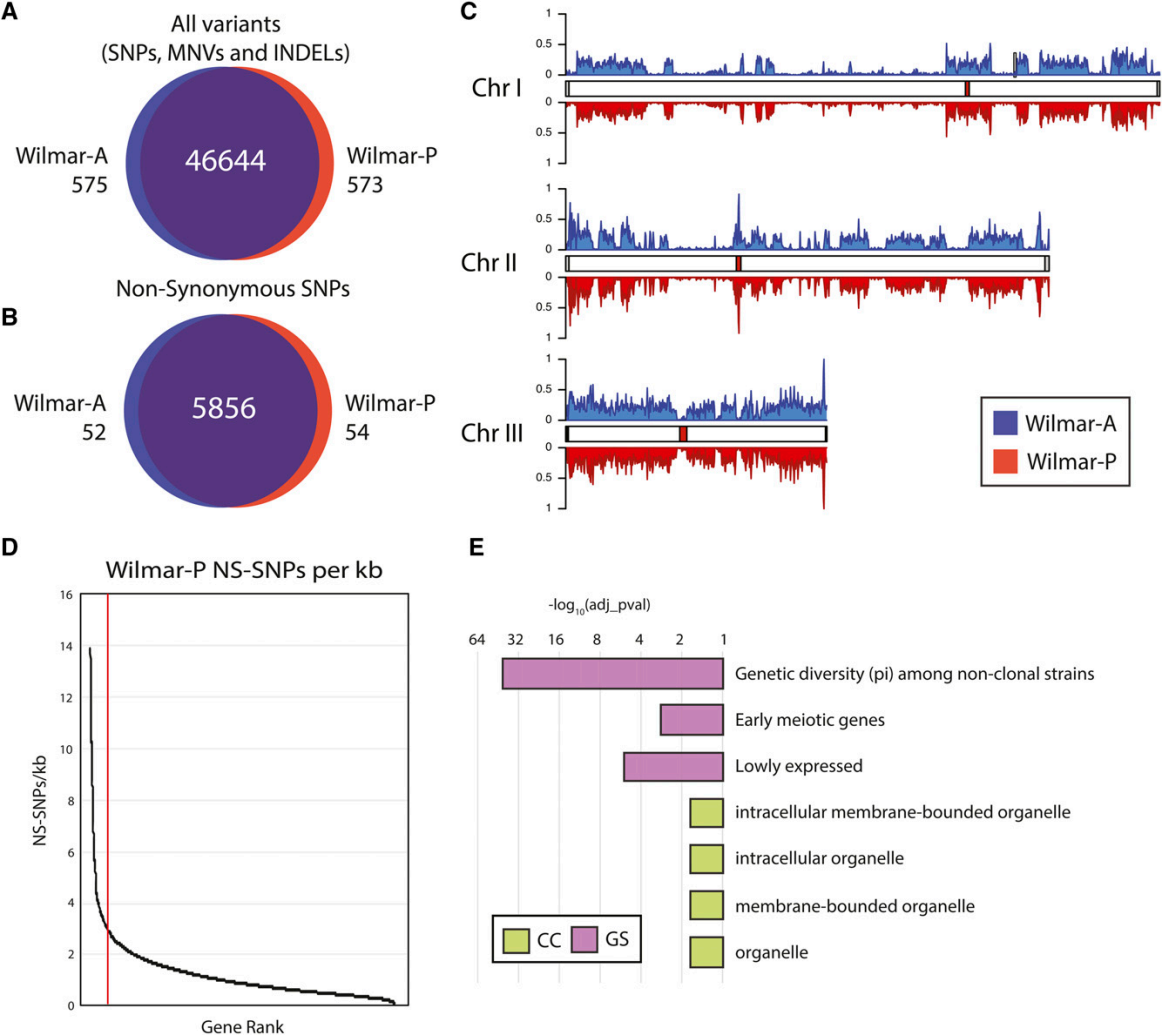
Almost half of laboratory *S. pombe* genes are affected by non-synonymous mutations in the Wilmar background. A) Venn diagram showing overlap of all variants (SNPs, MNVs and INDELs) detected in Wilmar A and Wilmar-P. B) Venn diagram showing overlap of non-synonymous SNPs (NS-SNPs) detected in Wilmar A and Wilmar-P. C) Density of Wilmar-A (blue) and Wilmar-P (red) SNPs across the laboratory *S. pombe* genome. D) Distribution of NS-SNPs per 1000 base pairs (NS-SNPs/kb) for each gene in Wilmar-P ranked in descending order. Red line indicates a threshold of 3 NS-SNPs/kb set for GO analysis. E) GO term and gene set enrichment analysis for the 120 genes within Wilmar-P containing 3 or more NS-SNPs/kb. X-axis shows negative log_10_ transformation of false discovery rate adjusted p-values displayed on a log_2_ scale. CC = Cellular component, GS = Angeli gene set.

To investigate the genetic variation underlying the Wilmar-P phenotypes, we sequenced the Wilmar-P and Wilmar-A genomes using Illumina short-read sequencing, achieving high-depth (∼200X) coverage of both genomes (Table S2). Wilmar-A and Wilmar-P reads were mapped to the laboratory *S. pombe* reference genome and single nucleotide polymorphisms (SNPs), multiple nucleotide variants (MNVs), and insertions and deletions (INDELs) in each Wilmar isolate were called using freebayes and the Genome Analysis Toolkit (GATK) Unified Genotyper (DePristo *et al.* 2011; Garrison and Marth 2012). Following depth and quality filtering, approximately 47,000 total variants (SNPs, MNVs and INDELs) were identified for both Wilmar-P and Wilmar-A (Table S3), with 97.5% of identified variants found in both strains (Figure 2A). We annotated the predicted impact of each variant using the variant effect predictor (VEP) (McLaren *et al.* 2016). VEP identified 16,469 and 16,521 SNPs within protein-coding regions in Wilmar-A and Wilmar-P respectively (Table S3). Approximately two-thirds of these SNPs were synonymous changes (10,559 in Wilmar-A, and 10,613 in Wilmar-P) with approximately 5900 non-synonymous SNPs (NS-SNPs) detected in each isolate (Figure 2B). Just 52 (Wilmar-A) and 54 (Wilmar-P) NS-SNPs were unique to either strain. These unique NS-SNPs occurred primarily in sequence orphans, pseudogenes, transposable elements or genes encoding cell surface glycoproteins (Table S4). No strain specific differences in SNP distribution were observed across the genome (Figure 2C). Thus, sequence level variation is unlikely to completely explain the observed phenotypic differences of Wilmar-P compared to Wilmar-A.

Overall, 2296 and 2301 genes in Wilmar-A and Wilmar-P, respectively, contained at least one NS-SNP (Table S3). Thus, almost half of the ∼5114 protein-coding genes in the *S. pombe* reference genome contained at least one NS-SNP in the Wilmar background, with many genes containing multiple NS-SNPs (Table S3). Corrected for gene length, most genes contained less than 3 NS-SNPs per 1000 base pairs (NS-SNPs/kb) (Figure 2D). Just 120 genes contained 3 or more NS-SNPs/kb in Wilmar-P. This set of genes was enriched for lowly expressed and early meiotic genes as well as membrane bounded organelle cellular components (Figure 2E). Interestingly, genes with increased genetic diversity (pi) among non-clonal strains were also enriched (Jeffares *et al.* 2015) suggesting SNPs in these genes are common among non-laboratory strains of *S. pombe*.

Jeffares and colleagues previously analyzed the genomes of 57 genetically distinct populations of *S. pombe* originating across 5 continents from a variety of environments including wine, cane sugar, tequila, grape juice, African millet beer and molasses (Figure S1A) (Jeffares *et al.* 2015). We combined the genome-wide SNP profiles of the Wilmar isolates with the Jeffares *et al.* (2015) dataset to determine the genetic relatedness of the Wilmar isolate within the context of this collection of wild *S. pombe* strains. Strains originating from Europe and America largely clustered according to their geographic origin while those from Asia, Africa and Australia were interspersed between these two major clusters. The Wilmar isolates clustered within a broader subset of European and American strains among multiple other strains isolated from molasses (Figure S1B, S1C). In contrast, the laboratory *S. pombe* strain (JB22) clustered with a separate clade of European isolates, consistent with its proposed Swiss origins (Fantes and Hoffman 2016; Hoffman *et al.* 2015). Overall, these findings suggest that while there are few unique sequence-level genetic differences between Wilmar-A and Wilmar-P, the genetic variation observed in comparison to laboratory *S. pombe* is largely due to the common genetic background of the Wilmar strain.

### Wilmar strain genomic rearrangements are concentrated at chromosome II subtelomeres

Non-laboratory strains of *S. pombe* were previously shown to possess extensive karyotypic variation (Brown *et al.* 2011; Jeffares *et al.* 2017). We performed pulsed field gel electrophoresis (PFGE) to examine the macro-genomic landscape of the Wilmar isolates. We identified an enlarged chromosome II in both Wilmar-A and Wilmar-P compared to laboratory *S. pombe* (Figure S2). To investigate these macro-genomic changes in greater detail we performed Pacific Biosciences RSII long read sequencing of the Wilmar-P genome and used this data for *de novo* genome assembly. We achieved a high quality assembly of the Wilmar-P genome (N50 = 4.69Mb), with chromosome length scaffolds obtained for all three chromosomes and a complete mitochondrial genome (Table S5). Consistent with our PFGE experiments, chromosome II was approximately 200 kilobases larger in the Wilmar-P assembly compared to the laboratory *S. pombe* reference genome (Table S5).

**Figure 3:**
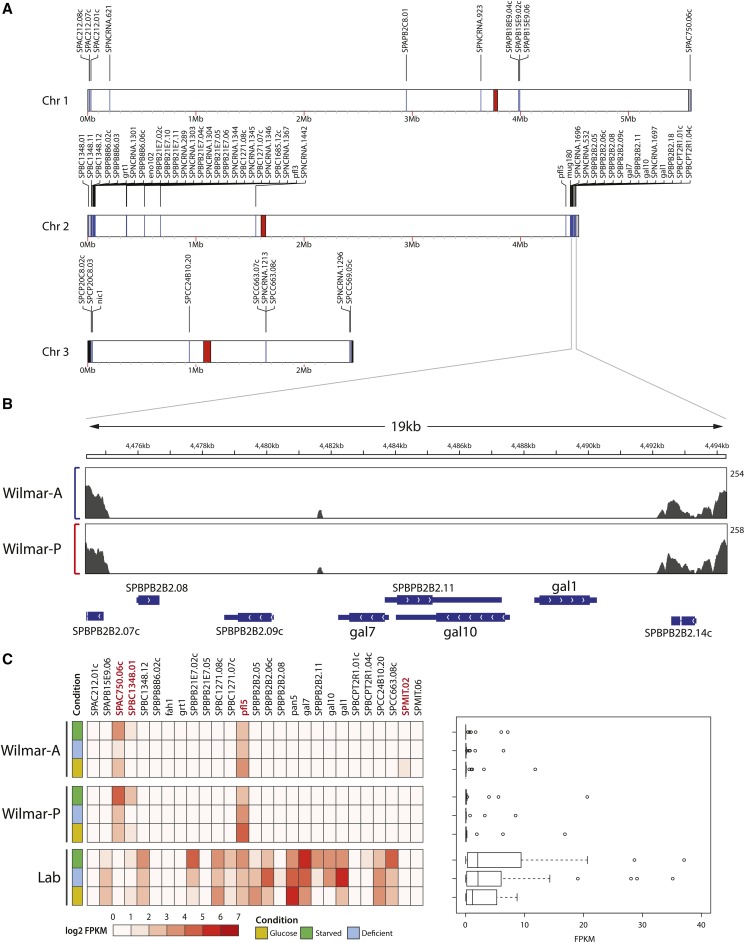
Gene loss in the Wilmar background is concentrated at chromosome II subtelomeres. A) Genome wide coverage map highlighting genes overlapping regions of zero coverage in both Wilmar-A and Wilmar-P. Red areas indicate chromosome centromeres. Blue lines indicate regions of genetic loss. B) Coverage plots of Wilmar-A (blue) and Wilmar-P (red) Illumina sequence data across a locus in the chromosome II subtelomere containing the galactose utilization gene cluster. Read depth scale indicated on the Y-axis where the minimum is zero. C) Left panel: Heatmap of FPKMs of genes overlapping regions of zero coverage in Wilmar-A (top), Wilmar-P (middle) and laboratory *S. pombe* (bottom) for the glucose, glucose-deficient (deficient) and glucose-starved (starved) conditions. Right panel: Boxplots showing FPKM distribution for the genes in the left hand side panel for each condition.

Pairwise chromosome alignments revealed two additional rearrangements in the Wilmar-P *vs.* laboratory *S. pombe* genome: a large inversion of the central region of chromosome I, which is common to many sequenced *S. pombe* isolates (Brown *et al.* 2011; Zanders *et al.* 2014), and a smaller inversion in chromosome II (Figure S3). Furthermore, we identified subtelomeric regions of laboratory *S. pombe* chromosome II which were missing from Wilmar-P genome (Figure S3). To investigate genomic loss events in the Wilmar background, we mapped the Wilmar-P and Wilmar-A Illumina reads to the laboratory *S. pombe* genome and searched for regions of zero coverage spanning at least 50bp. This identified 72 regions of the laboratory *S. pombe* genome, totalling ∼61kb, that were not represented in the Wilmar-A or Wilmar-P Illumina deep sequencing data (Table S6). No regions of genome loss were clearly unique to either Wilmar isolate, suggesting that differences in the total size of zero coverage regions between the isolates is due to subtle variation in either the raw sequence data or the read mapping results. Consistent with our chromosome alignments, the majority of zero-coverage regions were concentrated within the subtelomeric regions of chromosome II, while only small regions of zero-coverage were identified in chromosomes I or III (Figure 3A). Twenty seven zero-coverage regions overlapped an entire gene or the majority of a gene coding region suggesting these genes are absent or likely non-functional in Wilmar-A and Wilmar-P (Table S6). This gene set included the galactose utilization cluster members *gal1^+^*, *gal7^+^* and *gal10^+^* (Figure 3B).

To confirm the effect of these gene loss events on transcription, we performed RNA sequencing (RNA-seq) for Wilmar-A and Wilmar-P grown in glucose-sufficient (YES 3% glucose), glucose-deficient (YES 3% glycerol + 0.1% glucose) and glucose-starved (YES 3% glycerol) conditions and compared the expression of the coding regions overlapping regions of low coverage to previous RNA-seq data generated for laboratory *S. pombe* under the same conditions (Vassiliadis *et al.* 2019). With few exceptions, most genes predicted as lost showed no evidence of expression in Wilmar-A or Wilmar-P in any condition confirming their absence in the Wilmar background (Figure 3C). For some genes, expression in laboratory *S. pombe* was low across all conditions making comparison to the Wilmar isolates difficult. Overall these results suggest that significant chromosomal rearrangement events have occurred in the Wilmar strain lineage that are concentrated at chromosome II subtelomeres.

### Wilmar strain genomic rearrangement events include gain of Schizosacchaormyces octosporus sequences

**Figure 4:**
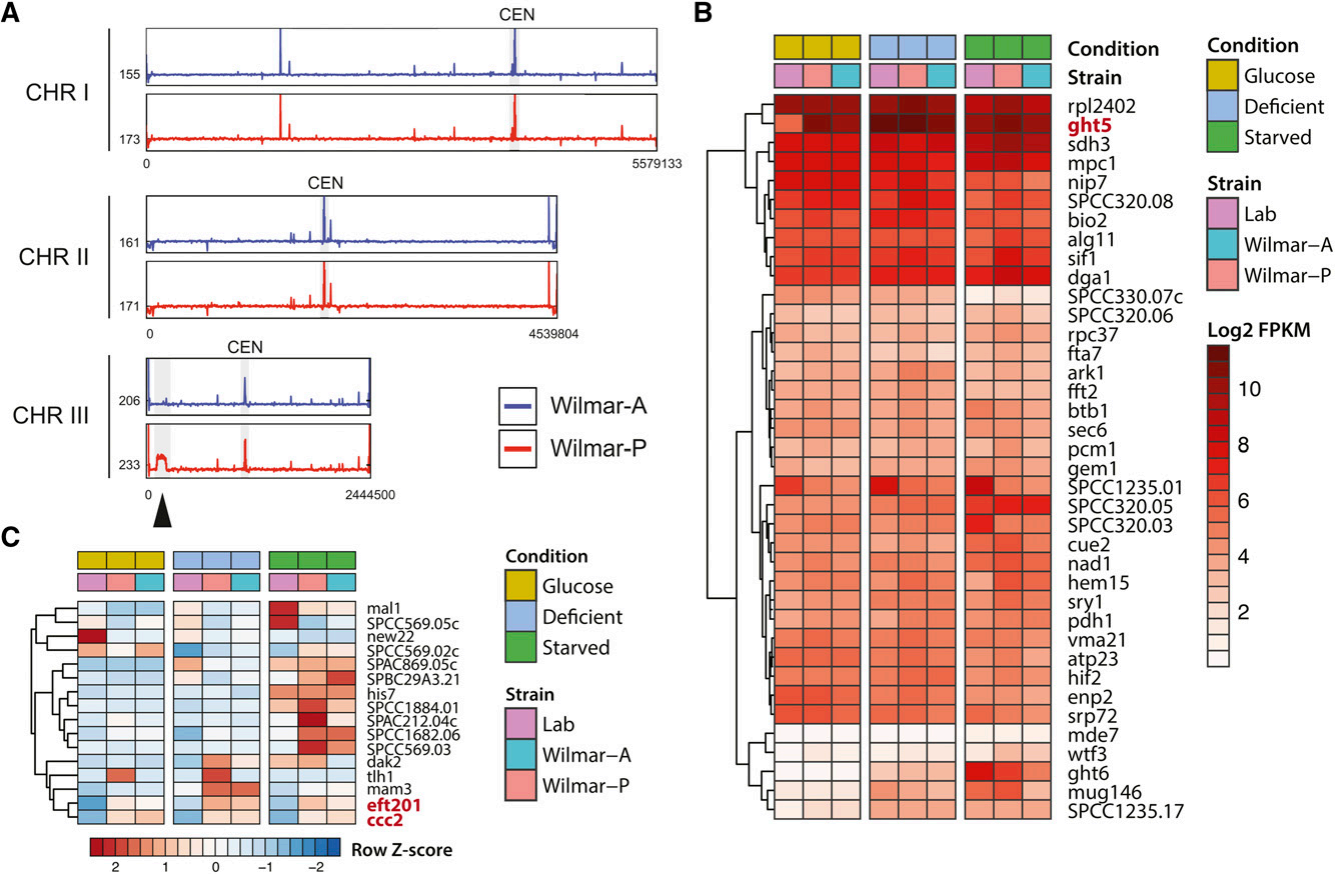
Gene gain in the Wilmar background includes a 100kb duplication unique to Wilmar-P. A) Illumina sequencing data depth of coverage plots for Wilmar-A and Wilmar-P across each chromosome of the laboratory *S. pombe* strain genome. The average read depth calculated using a 10kb sliding window with a 1kb step size across the chromosome is indicated. Y-axis indicates depth of coverage and is capped at 800. X-axis shows position within each chromosome with the total length of each in laboratory *S. pombe* indicated. Repetitive regions, including centromeric (CEN), telomeric and ribosomal DNA repeats are collapsed into single sequences within the laboratory strain reference genome sequence resulting in sharp increases in total read-depth at these locations due to reads from multiple repeats in the Wilmar strain genomes mapping to a single, collapsed sequence representing the repeat region. Smaller regions of increased read coverage likely indicate other repetitive elements such as transposons within the Wilmar strain genomes. One large region of approximately double the average chromosome read-depth, specific to Wilmar-P is shown in chromosome III (arrow). B) Heatmap of gene expression for the 38 protein coding genes within the Wilmar-P duplicated region across the glucose, glucose-deficient (deficient) and glucose-starved (starved) conditions. C) Heatmap of gene expression of other genes identified as amplified in the Wilmar background.

Our coverage analyses also revealed multiple areas of increased read coverage including a large duplication approximately ∼100kb in length in the subtelomeric left arm of chromosome III that was unique to Wilmar-P, suggesting it has arisen as a result of adaptation within the industrial environment (Figure 4A). This ∼100kb region contains 38 protein-coding genes, 12 annotated non-coding RNAs and a single transfer RNA encoding sequence (Figure S4, Table S7). Among the 38 protein-coding genes were six encoding transmembrane transporters, including the hexose transporter genes, *ght5*^+^ and *ght6*^+^. We examined the expression of these genes across our RNA-seq datasets and found that *ght5*^+^ was upregulated in Wilmar-P but not Wilmar-A suggesting that Wilmar-P may possess enhanced glucose uptake capability compared to Wilmar-A and laboratory *S. pombe* (Figure 4B). We examined copy number variation events in the Wilmar strain genome data and identified several additional regions of localized CNV (Table S8) including loci containing the copper transporter encoding gene *ccc2^+^* and the di-hydroxyacetone kinase encoding gene *dak2^+^* (Figure S5A, S5B). Identified regions of CNV were consistent across Wilmar-P and Wilmar-A. RNA-seq expression data of protein-coding genes within these CNV loci showed gene expression patterns for *ccc2*^+^ consistent with increased copy number across all growth conditions (Figure 4C) indicating increased copper import or efflux capability within the Wilmar background.

**Figure 5:**
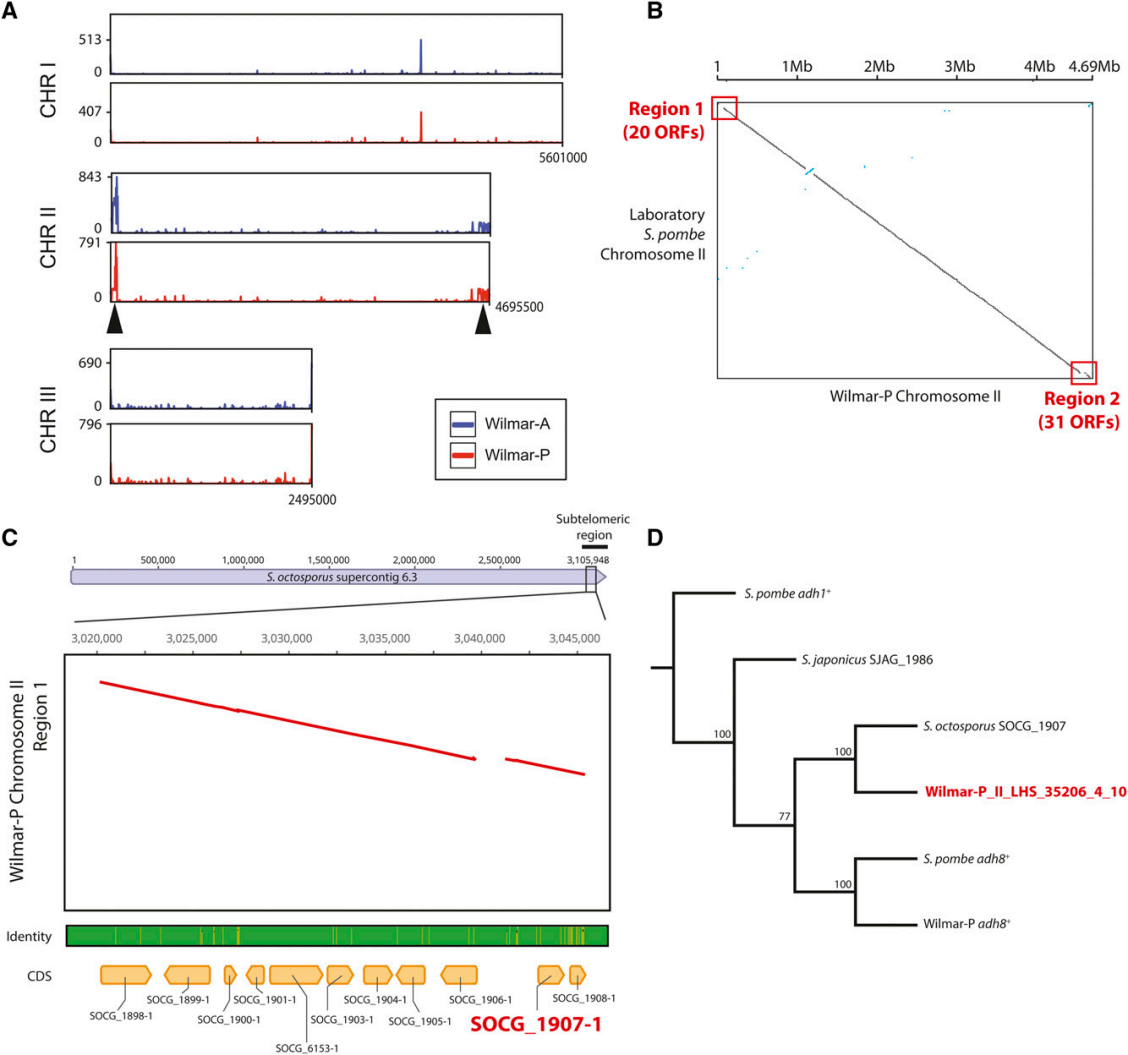
The Wilmar-P genome contains *Schizosaccharomyces octosporus* genes. A) Coverage plots of Wilmar-A and Wilmar-P Illumina reads that did not align to the laboratory *S. pombe* genome mapped back to the *de novo* assembled Wilmar-P genome. Average read depth calculated using a 10kb sliding window with a 1kb step size. Y-axis indicates depth of coverage and is capped at 900 (Wilmar-A) or 800 (Wilmar-P). X-axis shows position within each chromosome with the total length of each in the Wilmar-P genome. B) Whole chromosome alignment of Wilmar-P chromosome II *vs.* laboratory *S. pombe* chromosome II showing two regions unique to the Wilmar-P genome and the number of open reading frames (ORFs) detected within each. C) Whole chromosome alignment of *S. octosporus* supercontig 6.3 *vs.* Wilmar-P chromosome II Region 1. Map is centered on the region showing conserved synteny. Orthologous *S. octosporus* genes are labeled. The gene labeled in red, SOCG-1907-1, encodes an alcohol dehydrogenase orthologous to *S. pombe adh8*^+^ used in (D) to construct a neighbor joining tree. D) Tree generated using 100 bootstrap replicates with laboratory *S. pombe adh1*^+^ as the outgroup.

We next examined genetic sequences found uniquely in the Wilmar-A and Wilmar-P genomes. Approximately 4% of Wilmar-A and Wilmar-P Illumina reads did not map to the laboratory *S. pombe* genome. Of these unmapped reads, the majority (84% for Wilmar-A and 94% for Wilmar-P) could be mapped to the *de novo* assembled Wilmar-P genome, with read coverage primarily localized within the subtelomeric regions of chromosome II (Figure 5A) suggesting the novel sequences in the Wilmar background are primarily located in these regions. No regions appeared unique to either Wilmar-A or Wilmar-P. This observation was consistent with our CHEF and *de novo* assembly data (Figure S2B, Table S5). We identified 51 putative open reading frames (ORFs) with a minimum length of 500bp within the Wilmar-P specific chromosome II regions (Figure 5B). BLAST analyses returned hits for 44 of these ORFs with 22 matching laboratory *S. pombe* genes located in the subtelomeric regions of chromosome II which have likely undergone structural rearrangement in the Wilmar background (Table S9). Intriguingly, 22 ORFs possessed top BLAST hits from non *S. pombe* species, predominantly the related species *Schizosaccharomyces octosporus*. For most of these ORFs, the BLASTp and BLASTn identity to the respective *S. octosporus* sequence was >95%, whereas the nearest *S. pombe* ortholog was between ∼45–80% (Table S9).

We identified 10 *S. octosporus* ORFs syntenic with a region in *S. octosporus* supercontig 6.3, indicating that this region has likely originated from *S. octosporus* and not *S. pombe* (Figure 5C). These syntenic *S. octosporus* ORFs represented in the Wilmar-P genome encoded predicted alcohol dehydrogenase (SOCG_01907), elongation factor 1-gamma (SOCG_01903), arylsulfatase (SOCG_01906), aromatic amino acid transferase (SOCG_01904) and alpha-ketoglutarate dependent sulfonate dioxygenase (SOCG_01905) proteins (Table S9). Nucleotide alignment of the putative alcohol dehydrogenase sequence (Wilmar-P_II_LHS_35206_4_10) with orthologous sequences from *S. pombe* (*adh8*^+^), Wilmar-P (*adh8*^+^), *S. octosporus* (SOCG_01907) and *Schizosaccharomyces japonicus* (SJAG_1986) revealed the Wilmar-P sequence to be most conserved with *S. octosporus* SOCG_01907 (Figure 5D). We mapped the RNA-seq reads from Wilmar-A and Wilmar-P to the Wilmar-P genome to analyze the expression patterns of these *S. octosporus* sequences in the Wilmar background. Almost all identified *S. octosporus* sequences were expressed under glucose, glucose-deficient or glucose-starved conditions in both Wilmar-A and Wilmar-P with some displaying altered expression between conditions consistent with carbon-dependent transcriptional regulation (Table S10). These *S. octosporus* sequences identified in the Wilmar genome are possibly due to horizontal gene transfer between the two species or have been retained in the Wilmar strain genome since the divergence of *S. pombe* and *S. octosporus* lineages. Together, these results show that the Wilmar background has undergone considerable genomic rearrangement over evolutionary time, including copy number gain and loss and horizontal gene transfer of *S. ocotosporus* sequences, specifically at chromosome II subtelomeres, resulting in a net gain of genetic material compared to the laboratory *S. pombe* genome. Except for the chromosome III duplication unique to Wilmar-P, these genome scale rearrangements were also present in the Wilmar-A genome, suggesting that they were not induced by the molasses fermentation environment but were pre-existing in the Wilmar strain lineage.

### The Wilmar strain exhibits a distinct transcriptional program under laboratory growth conditions

**Figure 6:**
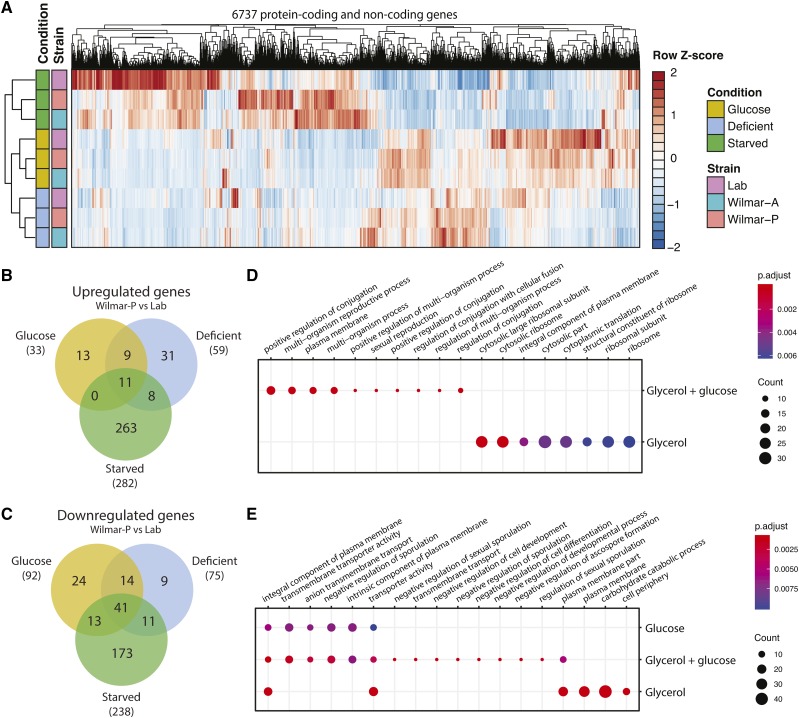
The Wilmar background exhibits a distinct transcriptional signature on laboratory media. A) Heatmap of global gene expression for 6737 protein-coding and non-coding genes in laboratory *S. pombe*, Wilmar-A and Wilmar-P in glucose, glucose-deficient (deficient) and glucose-starved (starved) conditions. Gene expression is presented as row normalized Z-scores. Venn diagram showing overlap of significantly upregulated (B) and downregulated (C) protein-coding genes in Wilmar-P *vs.* laboratory *S. pombe* in glucose, glucose-deficient (deficient) and glucose-starved (starved) conditions. Results of GO term enrichment for the upregulated (D) and downregulated (E) genes in Wilmar-P. Size of dots indicates number of genes in group. Only GO terms with a false discovery rate adjusted p-value < 0.01 are shown.

We next compared the transcriptional profiles of Wilmar-P and Wilmar-A to laboratory *S. pombe* cultured in laboratory media across each carbon condition. On a global level, a distinct transcriptional program was evident in the Wilmar isolates compared to laboratory *S. pombe* (Figure 6A). Differential gene expression analysis comparing Wilmar-P to Wilmar-A revealed few significant differences (Figure S6A-C) suggesting similar transcriptional programs are utilized by the two isolates under laboratory growth conditions (Figure S6D). Comparing Wilmar-P to laboratory *S. pombe*, a total of 285 protein-coding genes were significantly upregulated (Figure 6B) and 335 significantly downregulated (Figure 6C) in at least one of the three conditions tested (Table S11). Approximately 70% of these genes were differentially expressed only in the glucose-starved condition indicating fundamental differences in the transcriptional response of the Wilmar strain to carbon starvation (Figure 6A-C). In contrast the glucose and glucose-defiient conditions produced more similar transcriptional signatures (Figure 6A).

We examined enriched gene ontologies in the upregulated and downregulated subsets of genes in Wilmar-P from the three conditions. Genes encoding positive regulators of meiosis and sexual differentiation were strongly enriched in Wilmar-P relative to laboratory *S. pombe* in the glucose-deficient condition (Figure 6D, Table S12). In contrast, genes encoding ribosomal subunits and several transmembrane transporters including *caf5*^+^ (caffeine transport & drug efflux), *amt1*^+^ (ammonium transport) and *nic1*^+^ (heavy metal ion transport) were enriched in the glucose-starved condition. *S. pombe* is sensitive to nutrient deficient conditions and will undergo mating and meiosis in carbon or nitrogen deficient conditions (Mata *et al.* 2007; Vassiliadis *et al.* 2019). These results indicate that mating and meiosis pathways are more active in Wilmar-P growing in glucose-deficient conditions relative to laboratory *S. pombe*. In contrast, upregulation of ribosome and cytoplasmic translation genes in Wilmar-P in glucose-starved conditions suggests reduced sensitivity of Wilmar-P to normal starvation responses that occur during stationary phase and result in reduced translation rates (Ashe *et al.* 2000).

For the subset of downregulated genes in Wilmar-P compared to laboratory *S. pombe*, plasma membrane and transmembrane transport related genes were enriched across all three conditions (Figure 6E). In the glucose-deficient condition, genes involved in negative regulation of sexual differentiation, ion and anion transport were specifically enriched (Figure 6E, Table S12). In the glucose-starved condition genes involved in hexose transport and carbon catabolic processes were specifically downregulated reflecting reduced metabolic flux through canonical energy generating processes. Core environmental stress response induced genes were also significantly enriched (Table S12). Interestingly, several of the meiosis related genes both up- and downregulated in Wilmar-P in glucose-deficient conditions encoded cryptic Wtf proteins recently shown to encode dual poison-antidote meiotic drivers (Nuckolls *et al.* 2017; Hu *et al.* 2017). These genes show increased rates of DNA sequence polymorphism and copy number changes particularly in wild isolates (López Hernández and Zanders 2018). Thus, it is possible that expansion of the *wtf* family has occurred in the Wilmar strain lineage. Overall, our RNA-seq analysis revealed that the Wilmar background possesses a distinct transcriptional signature relative to laboratory *S. pombe* under glucose-sufficient, glucose-deficient and glucose-starved laboratory growth conditions.

### Wilmar-P adopts a stress tolerant transcriptional program under industrial fermentation conditions

**Figure 7:**
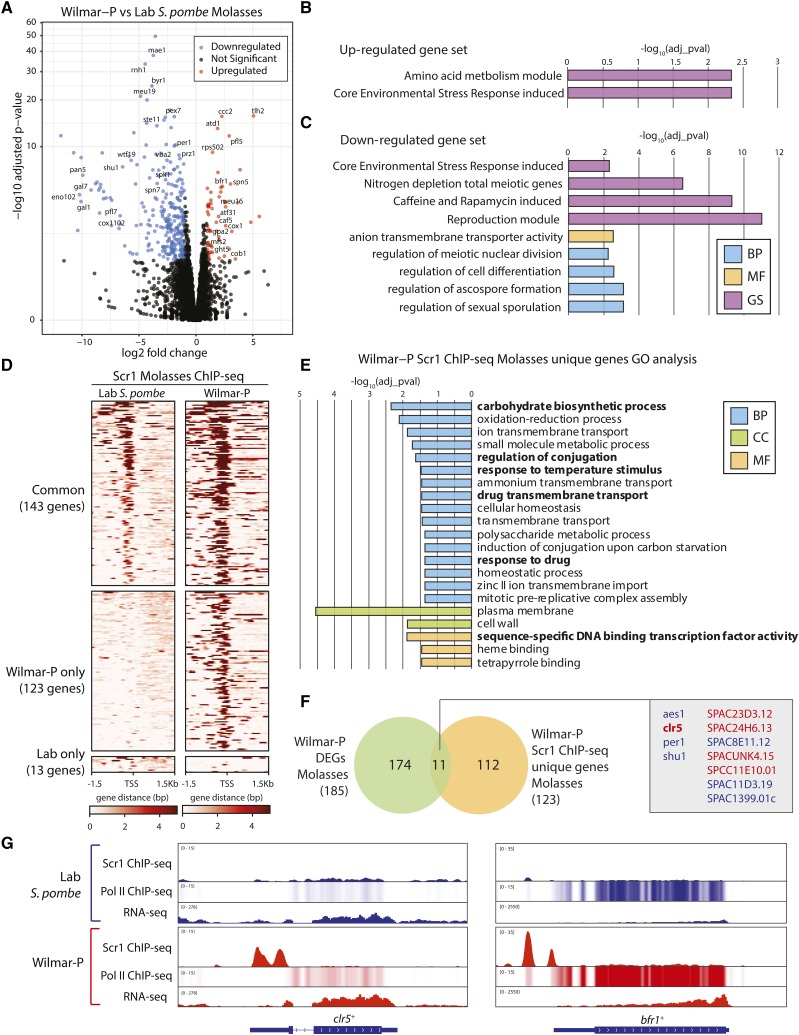
Wilmar-P installs a stress-tolerant transcriptional profile on molasses through the repurposing of Scr1 function. A) Volcano plot of differential gene expression in Wilmar-P *vs.* laboratory *S. pombe* on molasses. Genes of interest are labeled. Gene ontology and gene set enrichment analysis for the genes significantly upregulated (B) and downregulated (C) in Wilmar-P on molasses. BP = Biological process, MF = Molecular function, GS = Angeli gene set. D) Global heatmap of Scr1 ChIP-seq enrichment across gene transcription start sites (TSS) +/− 1.5kb in laboratory *S. pombe* and Wilmar-P in the molasses condition. Heatmap split by regions showing Scr1 binding in both strains (common), Wilmar-P only, or laboratory *S. pombe* only. E) Significantly enriched GO terms for the protein-coding genes bound by Scr1 only in Wilmar-P. BP = Biological process, CC = Cellular component, MF = Molecular function. F) Venn diagram showing overlap of genes significantly differentially expressed in Wilmar-P *vs.* laboratory *S. pombe* on molasses with protein-coding genes bound by Scr1 only in Wilmar-P. Genes within the overlap are labeled as upregulated (red) or downregulated (blue) in Wilmar-P. G) Genome browser views of Scr1 and PolII enrichment and RNA-seq normalized read coverage at the *clr5*^+^ and *bfr1*^+^ loci in laboratory *S. pombe* (blue) and Wilmar-P (red).

Next, we compared the transcriptional signatures of Wilmar-P and laboratory *S. pombe* cells cultured under molasses fermentation conditions. 185 protein-coding genes were significantly differentially expressed in Wilmar-P compared to laboratory *S. pombe* on molasses (Figure 7A). The majority (134/185, 72.4%) of these DEGs were downregulated suggesting that Wilmar-P primarily suppresses gene expression within the molasses environment (Figure 7A). Significant enrichment of cation and general transmembrane transporter encoding genes was evident among the molasses significantly upregulated genes (Figure 7B, Table S12). These upregulated genes included *ght5*^+^ and *ccc2*^+^, both duplicated in Wilmar-P, as well as *bfr1*^+^ and *caf5*^+^, two transmembrane transporters with known toxin efflux capability (Kawashima *et al.* 2012; Nagao *et al.* 1995; Turi and Rose 1995). The downregulated gene subset was enriched for core environmental stress pathway genes as well as genes involved in the regulation of cell differentiation and meiosis (Figure 7C, Table S12) suggesting that, in contrast to glucose-deficient conditions on laboratory medium, activation of these classical stress response pathways (Mata *et al.* 2007; Chen *et al.* 2003) in the molasses condition is repressed in Wilmar-P relative to laboratory *S. pombe*.

The RNA-seq data on laboratory media and molasses suggested improved carbon utilisation within the Wilmar-P strain consistent with our earlier growth assays (Figure 1B, 1C). Therefore, we further investigated the regulation of carbon metabolism pathway genes in Wilmar-P. Several previously defined targets of Scr1 (Vassiliadis *et al.* 2019), a transcriptional repressor of carbon source utilization genes in *S. pombe*, were among the differentially expressed genes in Wilmar-P on glucose or molasses (Figure S7A). To investigate potential alterations to the Scr1 regulatory network in Wilmar-P globally, we tagged Scr1 with a tandem affinity purification (TAP) tag in laboratory *S. pombe* and Wilmar-P cells, and performed chromatin immunoprecipitation sequencing (ChIP-seq) on laboratory *S. pombe* and Wilmar-P Scr1-TAP cells cultured in YES + glucose or molasses conditions. We also performed ChIP-seq against elongating RNA Polymerase II (PolII) to complement the RNA-seq data by specifically examining active transcription in the two strains grown on both conditions in case steady-state mRNAs measured by RNA-seq cannot reliably capture dynamic transcription changes (Tan and Wong 2019). No sequences unique to the Wilmar background were enriched for Scr1 (data not shown), so we analyzed the Wilmar-P ChIP-seq data following read mapping to the laboratory *S. pombe* genome.

Overall, the global binding profiles of Scr1 in the two strains was similar, with enrichment predominantly located upstream of gene transcription start sites in both the glucose and molasses condition (Figure 7D, S7B). Wilmar-P Scr1 binding on glucose largely recapitulated enrichment patterns previously defined for laboratory *S. pombe* Scr1 (Figure S7C, S7D) (Vassiliadis *et al.* 2019) however more binding sites were discovered in the molasses condition in both strains (Table S13, S14). Interestingly, we found an increased number of Scr1 binding sites in Wilmar-P compared to laboratory *S. pombe* in the molasses condition, with 266 Scr1 binding sites detected in Wilmar-P *vs.* 156 in laboratory *S. pombe* (Figure S7C). 143 protein-coding genes were bound by both laboratory *S. pombe* Scr1 and Wilmar-P Scr1. These genes were enriched primarily for carbon metabolism related gene ontologies similar to those previously identified for Scr1 in laboratory *S. pombe* cells in glucose sufficient conditions (Vassiliadis *et al.* 2019), suggesting the core regulatory roles of Scr1 are conserved in both strains in the molasses condition (Figure S7D). Just 13 genes were uniquely bound by laboratory *S. pombe* Scr1 on molasses. Among these genes were those required for galactose metabolism, *gal1*^+^, *gal7*^+^ and *gal10*^+^, which are absent in the Wilmar background (Figure S7E, Figure 3B). In contrast, 123 protein-coding genes which were bound only by Wilmar-P Scr1 were enriched for multiple stress response related ontologies including: response to drug, induction of conjugation and meiosis, response to temperature, ion transport and oxidation-reduction processes (Figure 7E). Multiple transcription factors were also present within this gene set including: *ace2*^+^, *esc1*^+^, *gaf1*^+^, *loz1*^+^, *pho7*^+^, *prr1*^+^, *rst2*^+^, *sfp1*^+^, *sre2*^+^ and *toe1*^+^, which function primarily in the regulation of nutrient and stress response pathways (Corkins *et al.* 2013; Kim *et al.* 2012; Alonso-Nuñez *et al.* 2005; Ohmiya *et al.* 2000; Higuchi *et al.* 2002; Kunitomo *et al.* 2000). Finally, 17 genes encoding transmembrane transporters were also present, including the iron ion transport and metabolism genes: *fet4*^+^, *frp1*^+^, *shu1*^+^, *str1*^+^ and *str3*^+^.

We compared Scr1 binding with changes in gene expression in Wilmar-P *vs.* laboratory *S. pombe* in the molasses condition and found that Scr1 was enriched at just 11 of the genes significantly differentially expressed in Wilmar-P in the molasses condition (Figure 7F). Of these 11 genes, 5 were significantly upregulated in Wilmar-P relative to laboratory *S. pombe* grown on molasses. One of these genes was *clr5*^+^, which is a negative regulator of meiosis related genes in *S. pombe* (Hansen *et al.* 2011). Scr1 was bound at the *clr5*^+^ promoter only in Wilmar-P and this was associated with increased transcription (as measured by RNA-seq and PolII ChIP-seq) in Wilmar-P, but not laboratory *S. pombe* (Figure 7G). We analyzed the expression patterns of all genes bound by Wilmar-P Scr1 in the molasses condition and identified 15 that showed significantly increased expression in Wilmar-P *vs.* laboratory *S. pombe*, despite the presence of Scr1 at the gene promoter (Table S15), suggesting that, contrary to its canonical role as a transcriptional repressor, Scr1 is associated with transcriptional activation at certain genes in Wilmar-P on molasses. One key example was the ABC transporter and efflux pump encoding gene, *bfr1*^+^, which confers increased growth in the presence of brefeldin A, a potent antifungal agent, when overexpressed and is associated with resistance to numerous other toxic agents (Akiyama *et al.* 2011; Iwaki *et al.* 2006; Nagao *et al.* 1995). Increased PolII enrichment was observed across *bfr1*^+^ in Wilmar-P compared to laboratory *S. pombe* gene as were higher mRNA levels (Figure 7G). *bfr1*^+^ was also significantly upregulated in Wilmar-P compared to laboratory *S. pombe* on molasses (Figure 7A). Interestingly, Scr1 enrichment in laboratory *S. pombe* was also observed at *bfr1*^+^, but was increased in Wilmar-P, suggesting that the presence of Scr1 at the *bfr1*^+^ promoter may have some role in its increased expression in Wilmar-P (Figure 7G). Since both Bfr1 and Clr5 are involved in the suppression of, and/or tolerance to stress responses in *S. pombe*, these results suggest that Wilmar-P Scr1 has acquired additional functions in the suppression of stress responses. However, whether Wilmar-P Scr1 acts as a transcriptional activator of these genes, or whether its presence facilitates the binding of other factors at the gene promoter to induce gene expression remains unclear and requires further investigation.

In summary, our results suggest that Wilmar-P is less susceptible to stressors within the molasses environment due to suppression of canonical stress response and sexual differentiation pathways and upregulation of transmembrane efflux pumps. Multiple stress tolerance related genes including *bfr1^+^* and *clr5^+^* were both directly regulated by Wilmar-P Scr1 in molasses and significantly upregulated compared to laboratory *S. pombe* suggesting reduced sensitivity to CCR mediated repression in the Wilmar background and/or the repurposing of Wilmar-P Scr1 function to promote gene activation.

## Discussion

*S. pombe* is a geographically diverse species and it is likely that untapped genomic variation exists within wild isolates that may be of value to industrial processes. Recent work has emerged in support of this hypothesis, with multiple laboratories reporting a striking array of genetic and phenotypic variation existing within geographically distinct populations of *S. pombe* (Brown *et al.* 2011; Fawcett *et al.* 2014; Jeffares *et al.* 2017; Jeffares *et al.* 2015; Benito *et al.* 2016). In this study we described the genomic and transcriptomic characterization of Wilmar-P, an *S. pombe* isolate that is utilized at industrial scale for the commercial production of bioethanol from sugarcane molasses. Molasses is a complex feedstock that presents multiple challenges to growth, including high sugar loading, osmotic pressure, contaminating microorganisms, residual toxins and/or chemicals from the sugarcane harvesting process, variable levels of base nutrients, minerals, and variable pH levels (Jain and Venkatasubramanian 2017; Wythes *et al.* 1978). Wilmar-P showed a significant growth advantage on molasses compared to Wilmar-A and laboratory *S. pombe* and exhibited resistance to increased temperature and the glucose analog 2-DOG, suggesting it has developed mechanisms that enable it to tolerate these pressures (Figure 1B, 1C). Previous studies have suggested that 2-DOG resistance is associated with altered hexose uptake (Novak *et al.* 1990) or upregulation of the *odr1*^+^ gene encoding an uncharacterized hydrolase (Vishwanatha *et al.* 2016). While *odr1^+^* expression was unchanged in Wilmar-A and Wilmar-P compared to laboratory *S. pombe* under the conditions tested in this study (Table S11), several hexose transporters exhibited altered regulatory dynamics. *S. pombe* contains eight hexose transporter encoding genes (*ght1-8*^+^) with *ght5*^+^ being the most highly expressed and important for viability under low glucose concentrations (Saitoh *et al.* 2015; Heiland *et al.* 2000). We found that *ght5^+^* was within a 100kb duplicated region unique to Wilmar-P (Figure S4) and was significantly upregulated in Wilmar-P across all carbon conditions examined (Figure 4B). We showed previously that Scr1, the key transcription factor driving CCR in *S. pombe*, represses both *ght5*^+^ in the presence of glucose (Vassiliadis *et al.* 2019). While Scr1 was bound at *ght5^+^* in glucose and molasses in Wilmar-P (Table S13), we observed significantly increased *ght5^+^* expression across both conditions compared to laboratory *S. pombe* (Figure 4B, 7A). Duplication of *ght5*^+^ might explain its increased expression, possibly improving the ability of Wilmar-P to acquire and metabolize sugars and so, potentially confering a metabolic advantage to this strain within the industrial environment.

Several smaller amplifications were present within both Wilmar-A and Wilmar-P. One of these amplified loci contained the copper transporting ATPase encoding gene *ccc2*^+^ (Figure S5A). Duplications of *ccc2*^+^ were also found in multiple geographically distinct *S. pombe* isolates (Brown *et al.* 2011). Like *ght5*^+^, *ccc2*^+^ was consistently upregulated in both Wilmar isolates compared to laboratory *S. pombe* across all carbon conditions tested, including molasses (Figure 4C, 7A). Copper is an important cofactor for many biological processes in eukaryotes, including aerobic respiration, iron homeostasis, detoxification, and assimilation of carbon and nitrogen sources (Kim *et al.* 2008), but in excess is known to be inhibitory to yeast growth and fermentation (Peña *et al.* 1998). In *S. cerevisiae*, loss of the *ccc2*^+^ homolog, *CCC2*, negatively affects both iron uptake and aerobic respiration (Yuan *et al.* 1995). Although the function of *ccc2*^+^ has not been extensively studied in *S. pombe*, additional copies may improve the ability of the Wilmar strains to withstand high concentrations of copper within molasses feedstocks, or to more efficiently acquire and/or utilize available copper within the industrial environment to maintain metabolic homeostasis.

Whole genome sequencing and *de novo* assembly of the Wilmar-P genome revealed extensive gene loss within chromosome II subtelomeres (Figure 3). This is consistent with previous studies that have identified karyotypic variation among genetically distinct populations of *S. pombe*, including extensive rearrangement of subtelomeric DNA (Jeffares *et al.* 2017; Brown *et al.* 2011; Bisht *et al.* 2008). Analysis of novel sequences within chromosome II subtelomeres in the Wilmar background identified a region containing 10 putative *S. octosporus* ORFs syntenic with those found in the native *S. octosporus* genome, suggesting that horizontal gene transfer has occurred between the two species somewhere along the Wilmar strain lineage (Figure 5C). Analysis of a putative *S. octosporus* alcohol dehydrogenase encoding sequence within Wilmar-P showed incongruency between the gene tree and the predicted *Schizosaccharomyces* species tree (Rhind *et al.* 2011), supporting the horizontal gene transfer hypothesis (Figure 5D). Ultimately, the genomic rearrangements observed in Wilmar-P support the hypothesis that genome plasticity, particularly at telomeres, may be a key mechanism driving increased genetic variation in wild *S. pombe* populations. It is also intriguing that the Wilmar-P regions containing *S. octosporus* genes originate from a subtelomeric region of *S. octosporus* supercontig 6.3 (Figure 5C). Jeffares and colleagues found 17 putative non-*S. pombe* genes among their 57 isolates with varying degrees of sequence identity to homologs in other Ascomycete fungi, however none of these occurred in syntenic blocks, or possessed such strong sequence conservation to other *Schizosaccharomyces* species genes as those observed in the Wilmar isolates (Jeffares *et al.* 2015). In contrast, several instances of novel genetic elements existing within industrial *S. cerevisiae* strains have been reported. For example, a novel 17kb DNA element predicted to encode 5 genes, including two transcription factors, is present in at least 7 wine/beer *S. cerevisiae* strains (Borneman *et al.* 2011; Novo *et al.* 2009). Phylogenetic and synteny analysis suggested that this cluster originated from *Zygosaccharomyces ballii* and is present in up to three copies within individual *S. cerevisiae* wine/beer strains (Borneman *et al.* 2011). Like the *S. octosporus* regions within the Wilmar background, genes within this cluster showed near-perfect nucleotide sequence identity to their *Z. ballii* orthologs suggesting that horizontal transmission of large DNA elements may be a mechanism by which novel genetic variation can be generated in microbial eukaryotes.

Our RNA-seq analysis revealed distinct transcriptional programs present in Wilmar-P and its ancestral isolate Wilmar-A relative to laboratory *S. pombe* (Figure 6A). In molasses, the industrially relevant environmental context, Wilmar-P showed significant upregulation of genes encoding the drug efflux pumps *bfr1^+^* and *caf5^+^* (Figure 7A). Overexpression of these genes is associated with brefeldin A and caffeine resistance respectively in *S. pombe* (Nagao *et al.* 1995; Benko *et al.* 2004). In contrast, loss of *bfr1*^+^ function results in sensitivity to a range of drugs including caffeine, brefeldin A, cerulenin, clotrimazole, cycloheximide, 4-nitroquinoline oxide and tributyltin (Iwaki *et al.* 2006; Calvo *et al.* 2009; Akiyama *et al.* 2011). It is unclear whether the molasses used by Wilmar Ltd. contains these, or other substances that might be inhibitory to *S. pombe* growth, however upregulation of *bfr1*^+^ and *caf5*^+^ potentially improves the ability of Wilmar-P to export toxic substances out of the cell and so contribute to the improved growth phenotype of Wilmar-P on molasses compared to Wilmar-A and laboratory *S. pombe*. ChIP-seq of Scr1 in Wilmar-P and laboratory *S. pombe* grown on molasses revealed significant enrichment of Scr1 at the *bfr1*^+^ promoter in Wilmar-P, but not laboratory *S. pombe* (Figure 7G). This enrichment was associated with both the increase in *bfr1*^+^ mRNA levels, and increased PolII enrichment. Overexpression of ATP-binding type cassette efflux transporters has been associated with increased tolerance of *S. cerevisiae* industrial strains to alkane biofuels and, more recently, to multi-drug resistance phenotypes in clinical isolates of *Candida albicans*, suggesting these transporters are key to the removal of toxic substances from the cell (Ling *et al.* 2013; Kell *et al.* 2015; Popp *et al.* 2017; Rocha *et al.* 2017).

Alongside upregulation of efflux transporters, the sexual differentiation response gene *ste11*^+^, and mitogen-activated protein kinase (MAPK) pathway genes, *byr1*^+^ and *spk1*^+^, were specifically downregulated in Wilmar-P compared to laboratory *S. pombe* on molasses (Figure 7A). Previous work in *S. pombe*, *S. cerevisiae* and *C. albicans* has shown that the MAPK cascade induces sexual differentiation in the presence of stress stimuli (Bahn *et al.* 2007). Thus, reduced expression of MAPK and sexual differentiation genes in Wilmar-P on molasses could maintain its ability to proliferate under conditions that are normally associated with sexual differentiation and meiosis in laboratory *S. pombe*. Given that Scr1 was not found at the promoters of *byr1*^+^, *spk1*^+^ or *ste11*^+^ in Wilmar-P on molasses, this downregulation could instead be caused by the silencing factor encoding gene *clr5*^+^ or any of the other stress response related transcription factors to which Wilmar-P Scr1 was uniquely bound in the molasses condition (Figure 7E, Table S13). Like *bfr1*^+^, *clr5*^+^ was significantly upregulated in Wilmar-P compared to laboratory *S. pombe* on molasses (Figure 7A). Moreover, Scr1 was bound to the *clr5*^+^ promoter in Wilmar-P on molasses (Figure 7G). Hansen *et al.* (2011) previously found a strong overlap between those genes regulated by Ste11 and Clr5, and that *ste11*^+^ itself is also a target of Clr5 mediated repression (Hansen *et al.* 2011). Taken together, our results suggest that Scr1 may directly play a role in this upregulation of *clr5*^+^ and, indirectly, the repression of Ste11 and other stress response genes in Wilmar-P in the molasses condition.

Stress responses are the result of a complex interplay between sensory and signaling machinery that are still not completely understood despite intense research across multiple eukaryotic systems. Wilmar-P has developed an improved ability to withstand these challenges in the molasses environment through genomic and transcriptomic adaptation including regulatory plasticity involving the Scr1 transcription factor. Considering that *S. pombe* is increasingly being explored for application in industrial processes, including winemaking (Benito *et al.* 2016; Benito *et al.* 2014; Mylona *et al.* 2016), bioethanol (Saithong *et al.* 2009; Choi *et al.* 2010; Abubaker *et al.* 2012), heterologous protein production (Idiris *et al.* 2006; Takegawa *et al.* 2009; Giga-Hama *et al.* 2007; Abe *et al.* 2009), drug discovery (Demirbas *et al.* 2011) and pharmaceutical production (Choi *et al.* 2017), it is of considerable interest to perform similar profiling across a range of industrial *S. pombe* isolates, as this may identify new strains suitable for application in the industrial setting. It is hoped that this work will provide impetus to further explore genomic and transcriptomic variation within *S. pombe* isolates and enable the metabolic/genetic engineering of “designer” strains, which possess improved industrial capabilities.
